# Advances in paper-based electrochemical immunosensors: review of fabrication strategies and biomedical applications

**DOI:** 10.1098/rsos.230940

**Published:** 2023-11-29

**Authors:** Jarid du Plooy, Nazeem Jahed, Emmanuel Iwuoha, Keagan Pokpas

**Affiliations:** SensorLab, Department of Chemistry, University of the Western Cape, Robert Sobukwe Road, Bellville 7535, South Africa

**Keywords:** paper-based devices, electrochemistry, immunosensor, antibody–antigen interactions, sensing

## Abstract

Cellulose paper-based sensing devices have shown promise in addressing the accuracy, sensitivity, selectivity, analysis time and cost of current disease diagnostic tools owing to their excellent physical and physiochemical properties, high surface-area-to-volume ratio, strong adsorption capabilities, ease of chemical functionalization for immobilization, biodegradability, biocompatibility and liquid transport by simple capillary action. This review provides a comprehensive overview of recent advancements in the field of electrochemical immunosensing for various diseases, particularly in underdeveloped regions and globally. It highlights the significant progress in fabrication techniques, fluid control, signal transduction and paper substrates, shedding light on their respective advantages and disadvantages. The primary objective of this review article is to compile recent advances in the field of electrochemical immunosensing for the early detection of diseases prevalent in underdeveloped regions and globally, including cancer biomarkers, bacteria, proteins and viruses. Herein, the critical need for new, simplistic early detection strategies to combat future disease outbreaks and prevent global pandemics is addressed. Moreover, recent advancements in fabrication techniques, including lithography, printing and electrodeposition as well as device orientation, substrate type and electrode modification, have highlighted their potential for enhancing sensitivity and accuracy.

## 1. Introduction

Cellulose, a versatile natural biopolymer, is integral to paper-based sensors. Its common derivative, paper, offers numerous physiochemical benefits like high surface area, strong adsorption and easy chemical functionalization. Its biodegradability and biocompatibility make it an excellent choice for green sensing [1] and energy [2] applications, and it enables capillary action for liquid transport [3,4]. Microfluidic paper-based analytical devices (µPADs) have revolutionized fluid handling, separation and analysis [5]. They excel as point-of-care (POC) devices, known for low-cost production, compactness, portability, rapid testing, high selectivity functionalization and low sample volume requirements. The sensitivity of the paper-based detection hinges on the affinity and transduction approach. Spectroscopic, colorimetric, biosensing, electrochemical and combined approaches are most used in the literature. Of these, electrochemical approaches have been widely employed for disease monitoring and biomedical research. The choice of electrode and its modifiers is a critical factor for consideration when combined with electrochemical methods [6]. Various electrode materials, including carbon, metal, polymer and nanoparticle-modified electrodes, are explored, enhancing paper-based substrates. Current research centres on electrode fabrication and substrate patterning techniques such as inkjet printing, wax printing, wet etching and screen printing [6,7].

Electrochemical paper-based POC devices involve chemical reactions that produce a measured electric current under a supplied voltage at paper-based substrates and have been found to be useful in the fields of chemistry and biosensors. Electrochemical sensors generally exist in four types—amperometric [8], conductometric [9], impedimetric and potentiometric [10]—and have expanded to field effect transistors (FET) [11] based on the transducer used. Electrochemical sensors of either type have shown success in a number of applications because of their need for low power input, ability to differentiate oxidation states of materials and monitor binding interactions, and cost-effective operation lending itself well to POC analysis.

**Figure 1.**
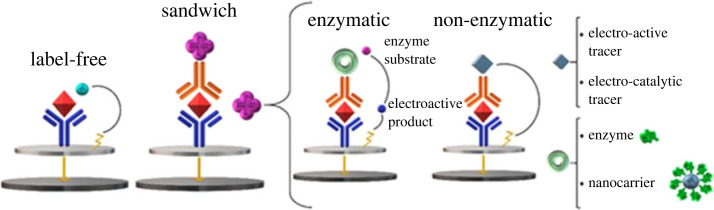
Examples of amperometric immunosensor configurations in direct, label-free, and enzymatic and redox-label sandwich approaches. Reprinted with the necessary creative commons license agreement of MDPI [16].

In the 1980s, Heinemannn *et al*. pioneered the development of electrochemical immunosensors (EIs), as reported by Rusling [12]. Since then, immunosensing has witnessed exponential growth. These sensors typically employ antibodies as a biorecognition element, selectively binding to target analytes. A transducer converts the antibody–antigen interaction into a detectable signal [13]. Combining immunosensors with electrochemistry has introduced new possibilities in both labelled and label-free configurations. Recently, EIs have gained traction due to their high selectivity interactions and ability to detect antigens at low concentrations in the nanogram per millilitre to microgram per millilitre range [14]. In particular, numerous attempts have been made to improve the biocompatibility, selectivity and cost of EIs through impregnation, 3D printing, integrated microfluidics and paper-based platforms [15]. These signals can be further amplified using nanoparticle enhancement, or enzyme- or DNA-based techniques [1]. EIs have proven invaluable in monitoring disease outbreaks, thanks to their highly sensitive and selective immunocomplex formation at the electrode surface [13]. Common targets include cancer, bacterial infections and other virus biomarkers as a means of early diagnosis. The exceptional selectivity of EIs arises from the interaction between antigens and disease-specific antibodies. Antibodies, shaped like ‘Y’ immunoglobulins (Ig), consist of peptide chains. They can be monoclonal or polyclonal based on the number of antigen-binding sites and used as a bioreceptor. Binding occurs through non-covalent van der Waals interactions following two configurations: (i) sandwich and (ii) direct via labelled and label-free methods, as shown in figure 1 [16].

Ongoing research is focused on improving the fabrication techniques of paper-based electrodes, enhancing signal amplification strategies and expanding the range of detectable biomarkers to address low-signal, resolution and sample preparation challenges.

This review provides an exploration of current trends in paper-based EIs. It covers the fundamental operating principle of EIs, techniques for patterning paper for electrode fabrication and the crucial aspect of hydrophobic barrier formation. Additionally, the review delves into the common methods of electrode modifications commonly used in EIs. A significant portion of the review is dedicated to examining recent advancements in detection methods, the selection of biomarkers and analytes, and the integration of nanoparticles for enhanced performance. The characterization techniques used for analysis are also discussed. Furthermore, the review addresses existing research gaps and evaluates the strengths and limitations of contemporary paper-based EIs. It provides valuable insights for future research directions in this rapidly evolving field, ultimately concluding the review.

## 2. Paper modification

Functionalizing paper-based substrates with specific reagents for electrodes, channels and reagent incorporation is crucial for the performance of EIs. Manipulating factors like the thickness, pore distribution and absorption rate of the paper substrates enables easy customization of µPADs for different applications [7]. Selecting the appropriate type of paper, such as chromatographic, filter, tissue, photographic, transparency, nitrocellulose or printing paper, is essential to match the intended application of the paper-based EIs. Common modification involves introducing wax and other hydrophobic chemical reagents to regulate fluid flow through hydrophilic channels formed by hydrophobic barriers in the woven paper matrix. Precise fluid control is vital for selective capillary wicking of reagents from high to low concentration regions within the system [17]. Various flow systems, ranging from straightforward to intricate, have been proposed recently. These include devices for flow, filtration, mixing and separation, as well as multi-step configurations that enable sequential reagent flow and washing to prevent contamination. Manipulating the rate and behaviour of reagent flow through channels of varying dimensions can be achieved by strategically placing hydrophobic and hydrophilic barriers. Different orientations, such as strip, stacked and folded, are used to govern transport through µPADs, limiting interactions between adjacent layers. Strip or lateral flow µPADs, widely used in analysis, offer excellent fluid control one-dimensional flow. Stacked and folded orientations address this limitation, enabling multi-dimensional flow and facilitating multiple simultaneous detections. However, misalignment of the reaction/detection layers during stacking or folding can lead to inaccurate results [18]. The surge in research on µPADs in recent years can be attributed to the cost-effectiveness and abundance of cellulose materials in nature. Paper's portability for onsite analysis, compatibility with various chemical, biochemical and medicinal applications, and its ability to transport fluids using capillary forces without the need for expensive machinery make it an invaluable component of analytical devices [19].

### 2.1. Fluid control

Efficiently directing the movement of reagents, electrolytes and other pertinent materials is a critical factor in the efficacy of paper-based microfluidic devices. The capability for capillary-driven liquid transport, obviating the necessity for external pumps, renders µPADs a compelling alternative to traditional microfluidic devices. Achieving this fluid control entails employing various engineering techniques, ranging from straightforward to intricate, and can be categorized into printing, cutting, light-assisted and other methodologies. Table 1 summarizes the advantages and disadvantages of the aforementioned fluid control techniques.

**Table 1.** Summary table of fluid control techniques.

fluid control technique	advantages	disadvantages	dimensions
photolithography	— produces narrow channel widths of 200 µm	— requirement of expensive and sophisticated equipment	— 0–200 µm
	— high precision of critical features in hydrophobic and hydrophilic barriers	— complicated procedure	
	— high resolution	— time-consuming	
plasma treatment	— flexible and bendable substrates can be modified	— expensive equipment	— <500 µm
	— eco-friendly approach	— requires various masks and templates for different fabrication patterns	
	— uses low-cost hydrophobic materials		
	— high resolution		
laser treatment	— high resolution	— smoke formation	— <1000 µm
	— reduction in fluid penetration	— channels formed restrict fluid flow; therefore requires extra coating	
	— low in cost compared to other high-resolution and -precision techniques		
	— narrow and small-featured channels		
wax dipping	— simple procedure	— only exposed surfaces are fabricated	— variable
	— low cost	— low resolutions	
	— can easily be implemented into etching processes	— inconsistent and unreproducible coatings	
	— can fabricate large devices		
stamping	— simple procedure	— complicated to produce stamps from hard materials	— <1000 µm
	— low cost	— low resolution	
		— inconsistent and unproducible coatings	
pen plotting	— simplistic procedure	— low resolution (by hand)	— variable
	— low-cost plotting materials (e.g. correction and permanent markers)	— time-consuming (by hand)	
	— high reproducibility (automated)	— inconsistent and unreproducible coatings (by hand)	
	— high speed and precision (automated)		
	— high resolution (automated)		
inkjet printing	— low-cost procedure	— reduces lifespan of standard printers	— 10–100 µm
	— minimal waste production	— low resolution (standard printer)	
	— high resolution (customized printer)		
	— no requirement of templates		
	— high speed for mass production		
flexographic printing	— great adaption to different substrates and materials	— only simplistic patterns	— <1000 µm
	— roll-to-roll production technique for mass production	— high cost of equipment	
		— constant maintenance of equipment	
		— time-consuming	
		— success dependent on smoothness of paper substrate	
wax printing	— simplistic and rapid	— requires machinery specifically designed for wax printing	— variable
	— large device fabrication		
	— low cost of materials		
	— prevents mixing of reagents		
	— no mask/templates required		

#### 2.1.1. Photolithography

Photolithography was the first technique used to pattern crucial features of µPADs with high precision [20–23]. This method allows hydrophobic barriers on paper substrates with sharp barrier angles [5,24] through patterning of paper using a photomask to a light-sensitive impregnated photoresist. However, due to its cost and complexity, its popularity has waned in favour of more cost-effective alternatives [25]. Moreover, the difficulty of preparing defect-free and accurate photomasks poses significant challenges. To address this issue, Asano & Shiraishi [26] introduced a µPAD for iron assays using a 3D-printed photomask and photolithography as a patterning technique. Octadecyltrichlorosilane *n*-hexane solution was impregnated in the chromatographic paper substrate prior to hydrophobization under UV light through a photomask to generate hydrophobic barriers. By contrast, Ma *et al*. [20] developed an eco-friendly, cost-effective photolithography-based method to obviate the use of a specialized UV exposure. In a more recent study, Beck *et al*. advanced UV photolithography for high-resolution hydrogel layer patterning. The *in situ* polymerization technique of different hydrogel polymers, UV exposure time and cross-linking density of the polymers were studied. The prepared devices were used in enzymatic microreactors for the first time [27]. Despite its advantages, photolithography faces challenges in solvent compatibility and barrier stiffness, which are destroyed by bending or folding. Nargang *et al*. [28] addressed this by using silanes patterned via photolithography, resulting in autoclavable barriers suitable for biological assays.

#### 2.1.2. Plasma treatment

Plasma treatment is an infrequently used technique for functionalizing paper-based microfluidics. It involves modifying the substrate material with ionized gas in a controlled vacuum chamber. This technique offers great potential as it does not alter the material's core properties, allowing flexible substrates. Additionally, it is capable of producing biocompatible components. Though not widely adopted, it shows promise for clinical diagnostics and other components in µPADs [29–31]. Li *et al*. [29] used an alkyl ketene dimer-heptane solution to hydrophobize paper for various functional components, including switches, microreactors and filters. Plasma treatment, in which the paper was sandwiched between patterned metal masks placed in a vacuum plasma reactor, facilitated hydrophobic patterning. However, it may have lower resolution and requires a custom metal mask [32]. Piccin *et al*. [31] explored using bio-derived polyurethane for microfluidic devices, achieving high relief through photolithography and nickel deposition. Oxygen plasma treatment further enhanced surface hydrophobicity.

#### 2.1.3. Wax printing

Wax printing has emerged as a cost-effective and efficient alternative to photoresists for fabricating µPADs [33] and has gained tremendous popularity. It is a straightforward two-step process: first, patterns are printed on paper substrates using solid wax, and then the patterned wax is heated to temperatures greater than its melting point, allowing the molten wax to penetrate the paper cross-section to create hydrophobic barriers, facilitating controlled fluid movement. This patterning results in hydrophilic channels, fluid reservoirs and reaction zones of desired dimensions in the µPAD responsible for liquid transport and storage. Moreover, wax printing can regulate reagent mixing and fluid flow rates [34,35]. Compared to other methods, wax printing is advantageous as it does not require specialized equipment or facilities, or extensive training, making it particularly suitable for resource-limited settings [36]. However, maskless applications may suffer from lower resolution due to uncontrolled wax melting and unwanted spreading [37,38]. Dungchai *et al*. [39] used a wax screen-printing method to fabricate a µPAD for the simultaneous determination of glucose and total iron in human serum samples. The authors controlled wax thickness by scrubbing it through a screen onto paper filters, resulting in adjustable channel dimensions between 650 µm and 1300 µm. Jang & Song [34] developed a facile flow-rate control method by varying the permeability with wax patterns. It was found that the brightness and length of the wax patterns could regulate dye flow rates. Similarly, Strong *et al*. [35] employed a printing technique for a more complex device with four flow channels, using wax printing to create delays and finely tune flow rates to the reaction zone. This allowed precise control of arrival times at the testing zone.

#### 2.1.4. Pen plotting

Using loaded cartridges for depositing hydrophobic inks and solutions offers a cost-effective alternative to automated and optical patterning techniques. Pen plotting allows controlled ink deposition onto substrates using correction and permanent markers, and polymer-loaded cartridges, creating hydrophobic and hydrophilic regions for fluid flow, incorporating impermeable fluidic breaks [40] but suffering from low resolution. While automated pen plotting achieves high-speed and precise patterning, manual approaches may be time-consuming. This technique has been applied in µPADs with variations in plotting ink, substrate and application [40–42]. Amin *et al*. [43] used a desktop pen plotter with custom multi-pen holder to fabricate hydrophobic barriers on µPADs, allowing simultaneous printing and enhancing efficiency. These µPADs were used for colorimetric urine assays to detect nitrite, urobilinogen, proteins, blood and pH [43]. While this technique demonstrated the first multi-head plotting for paper-based microfluidic devices, it introduced complexity due to the technique's design. The effectiveness of this technique depends on the durability of the substrate material during the patterning process, and the destruction of the paper substrate is a significant concern. Nuchtavorn & Macka [40] used pen plotting on Whatman filter paper No. 1, which has high sorption capabilities and is susceptible to deterioration. To enhance durability, they laminated the paper's underside before electronically controlled plotting. The polymer-based laminate bolstered the wetted device's structural integrity. Walia *et al*. [41] reported a novel pen-plotting method for µPAD fabrication using a simple writing technique. They modified a syringe by attaching a correctional pen tip and fitted it to the pen plotter, using BSA ink for patterning. Both techniques were successful two-step processes, but the choice between wax and BSA ink may hinge on the specific application. Wax requires higher temperatures to create hydrophobic paper, while BSA denatures at a lower temperature to achieve the same effect.

#### 2.1.5. Wax dipping

To address the limitations of wax printing, such as low resolution and inability to print on surfaces other than paper, wax dipping has been proposed. While simple, it can yield inconsistent films and coatings due to the variability in each dip. This process involves immersing the paper into molten wax to create hydrophobic barriers in exposed areas. Typically, it is combined with etching processes to refine the patterns by removing or modifying the deposited wax. Songjaroen *et al*. [44] introduced a novel approach to fabricated µPADs using wax dipping. They used an iron mould to protect specific areas from wax deposition, creating hydrophilic regions in various orientations. This technique was employed in a µPAD designed to separate blood plasma from whole blood and prevent contamination of the samples [45].

#### 2.1.6. Inkjet printing

Inkjet printing is a simplistic, template-free patterning method with minimal waste material for paper fabrication [46,47]. It uses a modified desktop or specialized printer [48] to deposit droplets of active materials of 10–100 µm on paper substrates, achieving high-resolution patterning. This technique has seen widespread use in electrical and sensing applications, and recent studies have explored its potential applications in various fields [49–51]. Unlike other printing techniques, inkjet printing stands out for its template-free, computer-assisted approach, allowing direct deposition of desired patterns without the need for a mask. Prabhu *et al*. [52] developed a low-cost POC device for diagnosing pathogenic fungi using inkjet printing of paraffin on paper. Paraffin penetrated the cross-section of the paper at elevated temperatures to create hydrophobic barriers with a thickness of 4 ± 1 µm and a hydrophilic channel width of 275 µm, which indicated good resolution and flow properties. In addition to hydrophobic barrier formation, recent studies have used inkjet printing to deliver functional materials with various properties for sensing applications. Li *et al*. [47] employed this technique to immobilize pH indicators on cellulose ester paper, enabling colorimetric pH detection without the use of hydrophobic barriers. On the other hand, Martins *et al*. [46] developed a strategy for better surface-enhanced Raman scattering experimentation by creating highly conductive and hydrophobic thin films using inkjet printing. The hydrophobic nanostructured surface, which combined silver and polystyrene nanoparticles through inkjet printing, helped concentrate the analyte in a small area on the hydrophobic paper substrate surface to prevent droplet spread, resulting in more sensitive surface-enhanced Raman scattering analysis through AgNP inclusion. As a result, interesting opportunities to direct other conductive and active materials via controlled or directed deposition pose interesting opportunities for paper-based sensing fabrication along with conventional flow device formation.

#### 2.1.7. Flexographic printing

**Figure 2.**
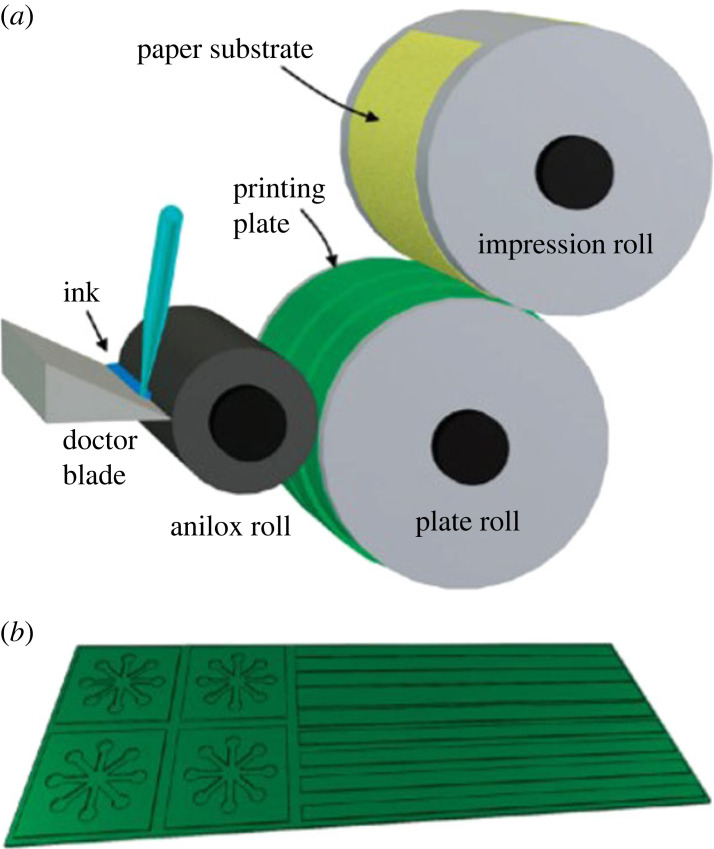
(*a*) Schematic diagram showing the flexographic printing setup used in the study. (*b*) Relief patterns in the green plate show the hydrophobic areas that will be formed on the paper. Reprinted with permission from [56]. Copyright (2023) American Chemical Society.

Flexographic printing is a relatively new direct printing technique used for fabricating hydrophobic barriers, coatings and ink patterns on µPADs [53–55]. It employs a flexible printing plate that prevents leakage and adapts well to different substrates and materials, enabling direct roll-to-roll production in existing printing machinery, as shown in figure 2. This makes it suitable for mass production without the need for heat treatment of the printed patterns. However, the high cost of equipment and limitations in printing complex designs have hindered its widespread adoption. Regular maintenance of machinery to avoid contamination and substrate roughness, and complex templates add to the high cost. Olkkonen *et al*. [56] demonstrated the use of flexographic printing with polystyrene in toluene to produce hydrophobic barriers on paper (figure 2). These barriers effectively penetrated the paper, resulting in leak-free structures of approximately 500 µm, which is slightly larger than those produced by inkjet printing and photolithography. This method has been validated for use in glucose monitoring. In another study, fluorene-based Schiff base inks were coated on paper substrates through flexographic printing by Muthamma *et al*. [54]. The developed ink exhibited blue-green fluorescence under UV light. The team suggested that this study has great potential for counterfeiting and electronic applications.

#### 2.1.8. Laser treatment

Laser treatment has emerged as a cost-effective and less complex alternative to photolithography and plasma treatment for fabrication [57]. It facilitates the production of small features with narrow barriers using a CO_2_ laser cutting/engraving machine [58]. Chitnis *et al*. [59] reported an inexpensive laser-treatment fabrication technique for creating a hydrophilic pattern on various paper types. The resulting features were approximately 62 ± 1 µm in size, exhibiting good resolution compared with other barrier fabrication methods. µPADs treated with microfluidic channels have been used to demonstrate chemical reactions using luminol-based haemoglobin detection [59]. Mahmud *et al*. [58] further advanced laser-treatment fabrication for producing compact and microscale features on µPADs. Their study showcased the potential for housing up to eight tests on a single device, creating a sophisticated paper-based detection system. The width of the hydrophobic barriers and features achieved through laser treatment was notably narrow, measuring approximately 39 ± 1.5 µm, which is significantly lower than other processes.

#### 2.1.9. Stamping

Stamping is one of the oldest and most direct fabrication techniques that use various stamps [60–62]. It has some drawbacks, such as producing low-resolution patterns and requiring complex processes for making hard stamps. Polydimethylsiloxane (PDMS) high-relief stamp, iron, steel and flash foam stamps (FFS) have been suggested, to date [63]. Dornelas *et al*. [64] developed a simple method for patterning PDMS barriers on µPADs using contact-printing low-cost rubber stamps. This involved printing a PDMS-hexane solution onto chromatographic paper using custom rubber stamps that held the desired patterns. Once the PDMS had penetrated the paper, it was cured to form a hydrophobic barrier. The best dimensions achieved using this method for the hydrophobic barriers and hydrophilic channels were 949 ± 88 µm and 771 ± 90 µm, respectively. Mathaweesansurn *et al*. [62] applied the stamping technique to create a customized µPAD for simultaneous multiple-point addition assays for creatine determination in human urine. They designed a custom stamp with eight reaction zones separated by hydrophobic barriers on Whatman filter paper No. 1, highlighting the versatility of stamp fabrication for various patterned shapes.

**Figure 3.**
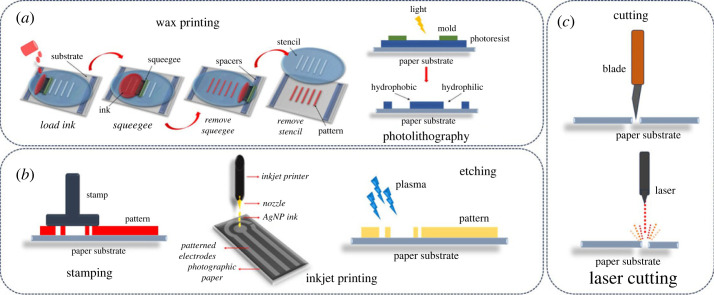
Schematic illustrations of selected (*a*) masked, (*b*) maskless and (*c*) cutting approaches for creating 2D µPADs. Copyright (2023). Redrawn with permission from [65].

An overview of various masked, maskless and cutting approaches for the fabrication of paper-based microfluidic devices is given in figure 3. The patterning techniques are subdivided based on the instrumentation requirements.

## 3. Design

### 3.1. Orientation of µPADs

Various orientations can be implemented in the µPAD design. The orientation selected gives the µPAD the best possible advantage and efficiency for the application and detection type. Examples of the various orientations found in µPADs include strip, folded (origami) and stacking orientations.

#### 3.1.1. Strip/lateral flow orientations

**Figure 4.**
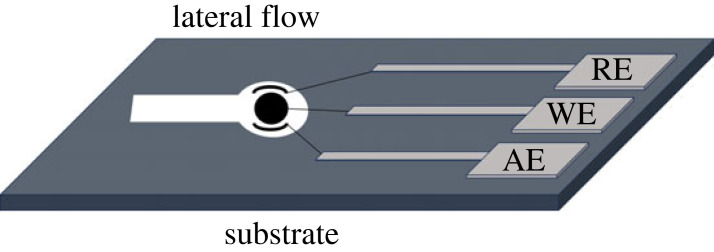
Schematic illustration of the lateral flow configuration of paper-based EIs.

Lateral flow channelling is easily achieved with the help of inkjet and wax printers, as well as the paper's permeability properties [66]. As previously mentioned, this orientation not only offers precise fluid control but also brings several benefits. Lateral flow µPADs are rapid, user-friendly without the need for pretreatments, cost-effective and stable, and have a long shelf life [67,68]. However, their speed can be affected by fluid viscosity, making analysis time dependent on the sample's nature. Such systems require only small sample volumes, an advantage, but accuracy in sample sizes is crucial for optimal sensitivity [67,69]. Lateral flow devices can have integrated electrode systems for electrochemical analyte detection [69–73]. This fluid transport to the electrodes is facilitated by capillary action. Srisomwat *et al.* [74] demonstrated an electrochemical lateral flow µPAD, for hepatitis B virus DNA detection, showcasing a two-dimensional (2D) µPAD example. Zhang *et al.* [72] conducted a recent study implementing lateral flow orientation for a rapid reverse transcript loop-mediated isothermal amplification assay diagnostic test for SARS-CoV-2. Using this orientation, they achieved results in under 40 min without the use of professional instruments and technicians. The diagnostic lateral flow assay had an accuracy of 100% in 12 synthetic and 12 clinical samples. An illustration of the lateral flow paper-based EI is given in figure 4.

#### 3.1.2. Folded/origami orientations

**Figure 5.**
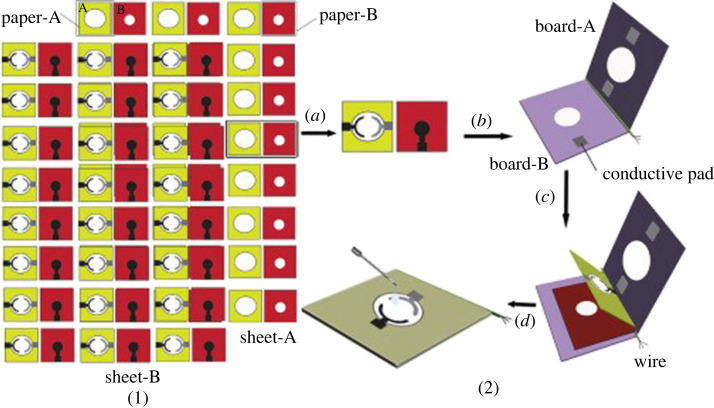
Schematic diagram showing the fabrication process and assay procedure of the electrochemiluminescence origami µPAD. Copyright (2023). Reprinted with permission from Elsevier [82].

The folded or origami orientation is a distinctive design feature of µPADs, allowing them to take on both flat and deployed complex orientations due to folds [75]. This design not only enables versatility but also facilitates efficient packaging and storage. In this orientation, gravimetric flow is typically employed to direct fluid towards the integrated electrode system, passing through reaction zones. Unlike one-dimensional flow in lateral flow µPADs, this approach eliminates reagent diffusion [76,77]. Folded orientations have found application in diverse fields including environmental monitoring, medical diagnosis and disease monitoring [78–81]. Yan *et al.* [82] developed a folded-based µPAD using electrochemiluminescence for carcinoembryonic antigen (CEA) detection. This immunosensor incorporated origami orientations, with the screen-printed working electrode modified for enhanced electron transport through gold and graphene. This represents an example of a three-dimensional (3D) µPAD, as shown in figure 5. Shen *et al*. [81] showed multiplex detection by creating 5-petal microfluidic channels as a bridge to portion samples on the paper. The goal of this µPAD was to create a biosensor capable of detecting various analytes in human body fluids, including urine, saliva and blood samples. Origami-oriented µPADs have seen significant advancements. Weng *et al*. [78] introduced a portable origami µPAD biosensor with a contemporary twist. The study applied the origami µPAD to monitor cortisol levels in human sweat, integrating smartphone-based analysis of the fluorescence emitted by the µPAD biosensing.

#### 3.1.3. Stacked orientations

**Figure 6.**
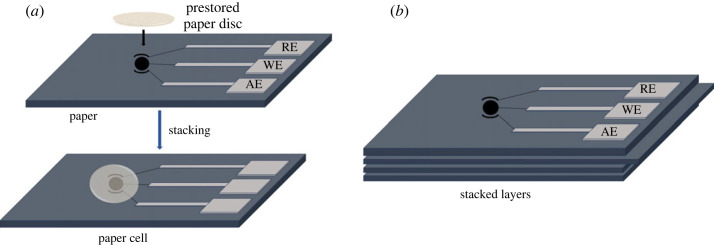
(*a*) Disc and (*b*) stacked orientations of 3D µPADs for EIs.

The stacked orientation is another example of a 3D µPAD, produced by layering multiple sheets of paper. This orientation addresses the drawbacks of one-dimensional flow and imperfect mixing or nonreactive areas in 2D paper configurations. Stacked orientations offer the advantage of multi-dimensional flow, which have various applications [83,84]. Unlike 2D orientations, these devices can distribute fluids for analysis into different test zones by channelling them vertically or horizontally [85]. Additionally, stacked orientations allow for layers to be separated, minimizing interactions and reducing non-reactive areas compared to 2D µPADs [86]. Carrilho *et al*. [33] conducted a detailed study on a simple and inexpensive fabrication method of µPADs using wax printing. They demonstrated a 3D µPAD constructed by a stacked orientation, where layers of patterned paper were bound together using double-sided adhesive tape. This approach showcased the µPAD's capability to distribute four individual samples from the top of the device into an array of 16 test zones at the bottom. Yukird *et al*. [84] illustrated the advantages of stacked orientations by developing a novel dual-detection µPAD for bisphenol A (BPA). This device achieved dual detection of the analyte through a stacked orientation that directs flow to opposite ends of the device via a lateral flow channel. The two detection methods used in this µPAD were electrochemical and laser desorption ionization mass spectrometry, improving the precision of BPA detection. In a separate approach, our group developed paper-based electrochemical cells (µPECs), cut from chromatographic paper for electrochemical monitoring of Ni-dimethylglyoxime complexes in water. The prepared µPECs were loaded with electrochemical reagents following a dry-storage approach and used in conjunction with commercial screen-printed electrodes in a stacked approach. The µPECs were easily tuned for a range of electrochemical applications [87]. Examples of the disc and stacked configurations are given in figure 6.

## 4. Paper substrate

Paper substrates are integral to the functionality of paper-based microfluidic, flow and electrochemical devices. With a history spanning over 2000 years, paper has served various purposes such as for writing, packaging, drawing and printing [88]. Cellulose, a key molecule found in paper, offers advantageous properties for research applications including porosity, pore size, wet strength, flow rate, stiffness, surface area and chemical reactivity [89]. The porous membrane of paper allows fluid flow by capillary action, guiding it to the desired reaction zones in µPADs [85]. The diverse pore sizes control the speed of fluid migration by capillary wicking. The specific paper type chosen is tailored to the desired application of the device. Examples encompass filter paper, chromatography paper, glossy paper, bioactive paper [90,91] and nitrocellulose paper [89]. A wide array of paper substrates has been suggested for paper-based EIs, leveraging their characteristic pore size, sorption capabilities, ability to be functionalized and biodegradable nature. The analytical performance of the device varied based on the cellulose matrix used. Filter and chromatography paper, particularly Whatman filter paper No. 1, is favoured in µPAD research for its ability to retain particles larger than 11 µm. While filter paper allows for high adsorption, it exhibits lower sensitivity for conductive materials. Glossy paper, composed of cellulose fibres and inorganic fillers [92], offers the advantage of easier manipulation of surface properties, such as smoothness. Hydrophobic photo paper, though unsuitable for flow systems, allows for easy surface functionalization, resulting in highly conductive paper sources. Nitrocellulose paper, featuring controlled porosity through cellulose nitration, excels in immobilizing macro-biomolecules, and binding proteins and nuclei acids [93,94]. Office papers have also been suggested but suffer from low sorption capabilities. All these paper sources provide eco-friendly substrate options with low-temperature operating requirements. The choice of paper, grade and fabrication approach is contingent on potential applications, emphasizing the need for optimization based on each application's requirements.

## 5. Electrode modification

Metallic and carbon electrodes are widely used in sensing of a range of analytes in paper-based devices and are responsible for applying potentials and carrying out oxidation-reduction reactions. Current research focuses on modifying the electrical conductor surface with different electrochemical functionalities and materials. Nanomaterials have emerged as the prime candidates for such electrode modifications. Their ability to augment the surface area of the electrode has made them highly sought after in recent years. This enhancement generally leads to increased sensitivity, improved electron transfer kinetics and amplified response signals for the electrode [95–97]. The electrodes commonly chosen for modification in paper-based sensing are typically carbon-based due to their availability and cost, including glassy carbon, carbon paste, screen-printed and pyrolytic graphite electrodes [98]. Several methods can be used to fabricate and modify electrodes using nanomaterials, such as drop-casting, screen printing, inkjet printing, electrode deposition and soft lithography. This section focuses on the widely used techniques for electrode modification.

### 5.1. Drop-casting

Drop-casting stands out as one of the simplest and most rapid techniques for depositing solid particulate at the electrode surface after the evaporation of the solvent to enhance electrochemical reactions [99]. This technique allows the deposition of a wide range of nano and other materials, each varying in shape and size [100]. The drop-casting process to functionalize the active working electrode area is used widely in modern research [101,102]. For instance, Pérez-Ràfols *et al.* [103] conducted a voltammetric experiment that simultaneously detected C(II) and Pb(II) using screen-printed electrodes. The authors modified the screen-printed electrode with Ag nanoparticles, which were drop-cast onto the electrode surface. This preparation method was performed using a 30 min drying time at 50°C. In another study, Shumyantseva *et al*. [104] investigated the electron transfer between cytochrome P450scc (CYP11A1) and Au nanoparticles. Both substances were drop-cast onto rhodium-graphite electrodes. In this study, cytochrome P450scc was incubated on the electrode overnight at 4°C. This shows that the incubation duration and temperature of a drop-cast substance vary depending on the substrate.

### 5.2. Screen printing

Screen printing is another effective method for preparing and modifying electrodes. It involves depositing various types of inks, which can be composed of nanoparticles, composites, enzymes or polymers, on plastic, ceramic or paper substrates. This enhances the sensitivity and selectivity of electrochemical sensing. The choice of ink depends on the specific application, with the fabrication process involving a conductive material that improves substrate adhesion and solvent compatibility [105]. The screen-printing process involved two main steps, namely infiltration of the ink through a mesh placed in the stencil, followed by the deposition of the ink on the substrate [106]. This results in a patterned electrode that corresponds to the stencil used. Ongoing recent advancements in screen-printed electrodes are expanding the range of available inks, leading to novel applications [107–109]. Ping *et al*. [110] developed a sensor for dopamine using an ionic liquid (IL)-modified screen-printed graphite electrode. They synthesized a composite of graphite-cellulose acetate to form a homogeneous and viscous ink. The ink was then deposited onto a PVC substrate. The resulting mixture was heated in an oven at 75°C for 30 min to allow complete solvent evaporation. Palisoc *et al*. [111] fabricated electrodes via a combination of screen printing and drop-casting, employing bismuth nanoparticle/Nafion composites. The team used an ultrasonicated PUR squeegee to print graphene ink onto a PVC substrate through a stencil mesh. This formed the working and counter electrodes, which were cured for 30 min at 130°C. The reference electrode was printed after the curing process and again cured for 30 min at 60°C.

### 5.3. Inkjet printing

Inkjet printing can also be used to deposit active materials in the form of an ink to an electrode substrate [112,113] without a stencil or squeegee, improving uniformity and accuracy [106]. Greater consideration should be taken for ink properties such as particle size, but fewer processing steps are needed because of the additive material deposition [114]. Piezoelectric inkjet printers, using transducers in the nozzle under high-voltage pressure, generate and release ink droplets. Different piezoelectric printers operate based on forces like shear, squeeze, push and bend. Inkjet printing is widely favored for fabricating electrodes, particularly in electrochemical sensors [92,115,116]. Kwon *et al*. [117] successfully dispensed carbon nanotube ink onto paper. Viviani *et al*. [118] studied the impact of carbon additives on the electrochemical performance of inkjet-printed thin-film Li_4_Ti_5_O_12_ (LTO) electrodes. This method obtained a resolution of 600 dpi, which was less than that reported in a previous study. Pokpas *et al*. [119,120] studied two approaches of carbon-nanomaterial-modified Ag nanoparticle electrodes for metal analysis in water.

### 5.4. Electrodeposition

Electrodeposition is a controlled process used to deposit a desired material onto a conductive surface using an electric current from an ionic solution [121]. Anodic or cathodic [122] processes can be used to modify the electrode surface with metals, ceramics, polymers, composites and nanomaterials [123]. Nanomaterials are often more useful in electrochemical sensing than their bulk counterparts [124–126]. The electrodeposition of nanomaterials onto electrodes results in enhancements in sensitivity and selectivity, which lead to specific recognition and pre-concentration of the analyte in question [127]. While electrodeposition offers advantages like producing high-performing coatings at low temperatures [128], it also has drawbacks like potential hydrogen embrittlement and the use of potentially toxic plating solutions [128]. Recently, Lorenzen *et al*. [126] produced an electrochemical impedance immunosensor for the detection of SARS-CoV-2 antibodies by electrodepositing PEDOT-AuNPs on the electrode. To stabilize the PEDOT/AuNP-modified electrode, 15 cyclic voltammetry (CV) cycles were performed. Another example is the work of Sánchez-Calvo *et al*. [125], who used hybrid nanomaterials, CNT-AuNPs and rGO-AuNPs, on the surface of a µPAD for mercury determination in environmental water via electrodeposition.

## 6. Paper-based EIs

Immunosensors are biosensors that rely on the affinity between antibodies and antigens, with a transducer converting this interaction into a measurable signal [13,14]. The signal corresponds to changes in the biomolecule's concentration [129]. Immunosensors are categorized into labelled and unlabelled. Labelled immunosensors introduce a detectable label on the immunocomplex, where the label is sensitively measured, while unlabelled immunosensors measure physical changes induced by the complex formation. Researchers enhance immunosensor sensitivity, performance and specificity by incorporating nanoparticles, quantum dots, enzymes, fluorescent labels and carbon materials [130]. EIs relying on voltammetry, potentiometry, impedance, electrochemiluminescence and others exhibit heightened sensitivity and selectivity for detecting various biological analytes [131]. Paper-based EIs are disposable in nature and allow for use in resource-limited settings with comparable performance. Nanoparticles incorporated into biosensors amplify the binding sites and enable efficient electron transfer. Here, paper-based EIs applied in biomedical diagnosis are discussed. Specific mention is made of biomarkers, bioreceptors, limits of detection, detection approach and electrode functionalization. Table 2 provides an overview of analytes detectable by microfluidic paper-based EIs in recent years, showcasing their versatility across various domains.

**Table 2.** Summary of recent work related to the application of paper-based EIs.

cause/disease	analyte	material and orientation	fabrication technique	nanomaterial	detection	detection limit	reference
oral cancer	Cyfra 21.1	photographic paper	pen on paper	Ag nano-ink	DPV	0.0025 ng ml^−1^	[132]
breast cancer	Claudin-7 and CD81	Whatman No. 1 filter paper	wax printing and inkjet printing	rGO	amperometry	Claudin-7: 0.4 pg ml^−1^	[133]
		2D lateral flow				CD81: 3 pg ml^−1^	
pancreatic cancer	PEAK1	Whatman No. 1 chromatography paper	manual screen printing	rGO and AuNPs	DPV	10 pg ml^−1^	[134]
liver cancer	AFP	Whatman No. 1 filter paper 2D lateral flow	photolithography and spin-coating	rGO and Au nanorods	SWV	0.005 ng ml^−1^	[135]
liver cancer	AFP	Whatman No. 1 chromatography paper 3D stacked	screen printing	Ag-GO nanocomposite	EIS	PBS: 1 ng ml^−1^	[136]
						Plasma: 10 ng ml^−1^	
ovarian cancer	CA 125	Whatman No. 1 chromatography paper 3D folded	screen printing	rGO/Thio/AuNP nanocomposite	DPV	0.012 ng ml^−1^	[137]
ovarian cancer	CA 125	photographic paper 2D	pen on paper	AuNP/Ag- rGO nanocomposite	chronoamperometry	0.94 ng ml^−1^	[138]
lung cancer	CEA	Whatman No. 1 filter paper 2D	wax printing and screen printing	NH_2_-GO/Thi/AuNP	DPV	0.01 ng ml^−1^	[139]
lung cancer	CEA	Whatman No. 1 chromatography paper 2D	screen printing	AuNP nano-ink	DPV	0.33 ng ml^−1^	[140]
oral/lung cancer	CEA and Cyfra 21.1	nanocellulose paper 2D lateral flow	lamination	quantum dot-doped polystyrene nanoparticle	/	Cyfra 21.1: 0.10 ng ml^−1^	[141]
						CEA: 0.35 ng ml^−1^	
breast cancer	CA 15-3	photographic paper 2D	inkjet printing	Ag/rGO ink + AuNPs	chronoamperometry	15 U/ml	[142]
pathogen (bacteria)	*S. aureus*	Whatman No. 3 chromatography paper 2D	stencil printing	SWCNT	DPV	13 CFU/ml	[143]
pathogen (bacteria)	*S. typhimurium*	filter paper 2D	drop-coating	PAMAM-NH_2_ dendrimer	potentiometry/EIS	5 cell/ml	[144]
pathogen (bacteria)	microcystin-LR	filter paper 2D	dip and dry	nanobiochar particles	amperometry	0.0017 µg l^−1^	[145]
pathogen (virus)	H1N1 influenza virus	Whatman No. 4 chromatography paper 2D	stencil printing	Silica NPs and SWCNT	DPV	13 CFU/ml	[146]
pathogen (virus)	H5N1, H7N9 and H9N2 influenza virus	Whatman No. 4 filter paper 2D	wax printing, screen printing and drop-casting	SWCNT	DPV	H5N1: 55.7 pg ml^−1^	[147]
						H7N9: 99.6 pg ml^−1^	
						H9N2: 54.0 pg ml^−1^	
pathogen (virus)	hepatitis B and C	Whatman No. 1 filter paper 3D folded	wax printing and CO_2_ laser treatment and screen printing	graphene	chronopotentiometry	hepatitis B: 18.2 pg ml^−1^	[148]
						Hepatitis C: 1.9 pg ml^−1^	
pathogen (virus)	SARS-CoV-2 (RBD S-protein)	cellulose-fibre pads 2D	screen printing	graphene/carbon ink	EIS	0.25 fg ml^−1^	[149]
pathogen (virus)	SARS-CoV-2 (RBD S-protein)	Whatman No. 1 filter paper 3D folded	wax printing and screen printing	cellulose nanocrystal and GO	DPV	2 fg ml^−1^	[150]
pathogen (virus)	SARS-CoV-2 (IgG and IgM antibodies)	Whatman No. 4 chromatography paper 3D folded	wax printing and screen printing	GO	SWV	IgG: 0.96 ng ml^−1^	[151]
						IgM: 0.14 ng ml^−1^	
pathogen (virus)	SARS-CoV-2 (RdRP gene)	Whatman filter paper	sputtering deposition	Au@CD core–shell NPs and graphite nanocrystals	DPV	0.15 pM	[152]
pathogen (virus)	SARS-CoV-2 (N gene)	Whatman filter paper 3D folded	wax printing and screen printing	/	amperometry	1 pM	[153]
inflammation	C-reactive protein	Whatman No. 1 filter paper 3D folded	screen printing	graphene/AuNPs	EIS	15 ng ml^−1^	[154]
inflammation	C-reactive protein	Whatman No. 1 filter paper 3D folded	screen printing and drop-casting	AuNPs	DPV	1.6 ng ml^−1^	[155]
pathogen (bacteria)	human IFN/*γ* (tuberculosis)	Whatman No. 1 filter paper 2D	screen printing	PANI-graphene	EIS	3.4 pg ml^−1^	[156]
protein (hormone)	17β-estradiol	Whatman No. 1 chromatography paper 2D lateral flow	wax printing and screen printing	MWCNTs/AuNPs	CV/DPV	10 pg ml^−1^	[157]
pathogen (fungus)	Aflatoxin B1	adhesive paper	cut printing and drop-casting	graphene/MWCNTs	EIS	0.62 ng ml^−1^	[158]

### 6.1. Immunoassays and immobilization chemistries for immunosensors

Immunoassay technologies, pivotal in clinical diagnosis, have seen significant advancements. Their evolution and automation make them increasingly viable for continuous monitoring [159] in various applications, like food quality control, and environmental and disease monitoring. Immunoassays work on the basis of binding antibodies (‘Y’-shaped protein) to specific sites (epitopes) on a target antigen [160], creating a highly repeatable and specific biosensor format. In heterogeneous assays, the primary antibody is affixed to a solid support, like an electrode, allowing the binding of the target antigen for recording a response [161]. Antibodies can be immobilized using passive adsorption/passivation or crosslinker mediation [162]. Passive adsorption is simple and lacks control over the antibody orientation, potentially limiting binding of antigens and reducing sensitivity. Crosslinker mediation involves the use of chemicals that contain a reactive centre at each terminal that allows for two functional groups to bind through the –NH_2_, –COOH and –SH functional moieties. Surface or antibody modification potentially leads to biomolecule activity loss. Several immunosensors have emerged, whereby biochemical, physical or enzymatic interactions have been employed to fulfill their specific function [163]. The resulting immunocomplex generates signals commonly detected through sandwiched or direct assays. Label-free immunosensors directly assess physical changes induced by the formation of an immunocomplex [164]. Introducing labels like enzymes, graphene, nanotubes, magnetic nanomaterials, noble metal nanoparticles, quantum dots, electroactive materials and luminescent materials enhances detection sensitivity and signal amplification [165–167]. Using these two methodologies, electrochemical techniques are employed to produce a variety of EIs for an array of target analytes.

### 6.2. Microfluidic paper-based electrochemical device (µPED) immunosensors for cancer biomarkers

Cancer affects the human body [168] through uncontrolled cell growth affecting the surrounding organs [169]. It is categorized into four main types—carcinoma, sarcoma, leukaemia and lymphomas [170]—which costs $895 billion (2015) per year to treat [171]. µPAD immunosensing of cancer biomarkers, which are biological molecules produced by the body in infected cancer patients [172], has increased considerably in recent years. Prostate-specific antigen (PSA), alpha-fetoprotein (AFP), CEA, cancer antigen (CA) 125 and 15-3, pseudopodium-enriched-atypical kinase one (PEAK1), and serum cytokeratin fragment 21.1 (Cyfra 21.1) offer excellent biorecognition of prostate, liver, lung, ovarian and breast, pancreatic, and oral cancers, respectively [173–176]. An overview of µPADs for diagnosis of cancer biomarkers is provided. The influence of paper substrates, nanomaterials and methodologies on the high sensitivity, specificity, reproducibility, detection limits, fast response times and low cost of production/affordability is provided [177]. Strategies to improve the shelf life, reuse and other critical parameters for cancer biomarker immunosensing are summarized. Challenges related to the stability of biomolecules are delaying the mass production of µPAD cancer biosensors [177].

Cyfra 21.1, a soluble fragment released during programmed cell death, is a biomarker for the early detection of various epithelial cell cancers such as oral cancers. Tofighi *et al*. [132] produced a paper-based EI for the determination of Cyfra 21.1 biomarker in human saliva using DPV. The sensor produced a limit of quantification of 0.0025 ng ml^−1^, well below the 3.8 ng ml^−1^ and 17.56 ng ml^−1^ for Cyfra 21.1 in uninfected human saliva and oral cancer patients, respectively, and offered improved sensitivity over existing electrochemical immunosensing approaches. The analyte was detected using pen-on-paper technology, where a synthesized Ag nano-ink with good physiochemical properties was handwritten on photographic paper to create a 3-electrode system capable of improving sensitivity, selectivity and stability. To establish covalent attachment of the Ag nano-ink sensing zone to the label-free anti-Cyfra 21.1 antibody, the –COOH groups of the working electrode were activated via NHS and EDC with good binding affinity. Chen *et al*. [141] used a different approach in which they constructed a lateral flow assay, which was not electrochemical but could detect both Cyfra 21.1 and CEA via the use of quantum-doped nanoparticles. This is an example of a fluorescent µPAD that could be an alternative to EIs.

Breast cancer remains a leading cause of cancer-related deaths in women globally, but it may be mitigated by early detection. Detection of circulating proteins, including CEA and cancer antigen 15.3 (CA 15-3), has shown promise to meet this end [133,142]. Hassanpour *et al.* [178] developed a paper-based immunoassay for breast cancer-specific carbohydrates (CA 15.3) with promise for future application in paper-based microfluidics. The study relied on the creation of a silver-decorated reduced graphene oxide (Ag-rGO) nanocomposite ink as an electrode material, hand-drawn on photographic paper substrates in conjunction with cysteine-capped gold nanoparticles as a signal amplification approach via chronoamperometry. Consequently, this work demonstrated good selectivity and specificity toward CA 15.3 determination in the presence of immobilized antibodies (anti-CA 15-3 Ab). The fabricated device suffered from a low sensitivity with an LLOQ of 15 U ml^−1^ when compared with other electrochemical sensors. Recently, extracellular vesicles have been proposed for early detection of breast cancer. Ortega *et al*. [133] addressed this problem by conducting a study on a sandwich-type µPAD EI for the dual amperometric determination of Claudin-7 and Cluster of Differentiation 81(CD81) breast cancer biomarkers [133]. The label-free immunosensor had a limit of detection of 0.4 pg ml^−1^ and 3 pg ml^−1^ for Claudin-7 and CD81, respectively. Two electrochemical cells, separated by microfluidic channels, were printed on opposite ends of Whatman No. 1 paper with individual electrode systems using GO ink reduced to rGO and silver ink, similar to Hassanpour's study, for the working and auxiliary electrodes, respectively. The sandwich was constructed by anti-Claudin-7 and -CD81 antibodies immobilized in channels on either side of the immunosensor, followed by HRP-labelled biomarkers. In the presence of HRP as a catalyst, the H_2_O_2_ added was reduced, allowing the TBC to be oxidized to benzoquinone for detection.

Pancreatic cancer is not usually detected until it progresses to an advanced stage. This is because of the lack of research on biomarker detection for the early diagnosis of pancreatic cancer. For the first time, Prasad *et al*. [134] developed a µPAD EI for the detection of a new biomarker, PEAK1, for the early detection of this cancer. The chromatographic substrate µPAD working electrode was modified with GO, followed by a sandwich construction of anti-PEAK1 antibody, where the PEAK1 biomarker was attached, and topped off with an AuNP-tagged-anti-PEAK1 antibody. For the activation of the –COOH groups on GO, NHS and EDC were used to treat the GO-paper substrate. DPV was used to measure the electrochemical response of the K_3_Fe(CN)_6_ reduction. The more concentrated the sample, the more AuNP-tagged-anti-PEAK1 antibodies were detected, resulting in a stronger electrochemical response due to the AuNPs. This immunosensor achieved high sensitivity with a low detection limit of 10 pg ml^−1^ and a linear range between 10 and 10^6^ pg ml^−1^. This work provides the only recent work conducted on pancreatic cancer detection using paper-based EIs.

Cancer is a fatal disease that continues to persist worldwide. Biomarker detection has become relevant in today's research, as early detection of tumour biomarkers can save many lives. A range of tumour biomarkers has been investigated for cancer biosensing. Of the wide range, only a handful have been employed for µPED analysis. AFP is the first serological biomarker for hepatocellular carcinoma (liver cancer) and birth defects in developing babies in pregnant women. Cao *et al*. [135] developed a novel disposable µPED immunosensor that detects for AFP in the 0.01 ng ml^−1^–100.0 ng ml^−1^ range. Whatman No. 1 filter paper SPCEs were modified using a reduced GO-tetra-ethylene pentaamine/Au nanocomposite (rGO-TEPA/Au) to anchor the capture antibodies at the μ-PEI surface. The rGO-TEPA enhanced the current response drastically because of its high conductivity, large surface area and increased stability, while AuNPs were incorporated to improve sensor biocompatibility. This combination produced an SPCE/rGO-TEPA/Au nanocomposite. The AFP-Ab_1_ immunocomplex was dropped onto the electrode. A signal probe, HRP-gold nanorod-signal antibody (HRP-GNR-Ab_2_), was dropped onto the modified SPCE to form an immunocomplex [(HRP-GNR-Ab_2_)- AFP-Ab_1_]. This immunosensor complex is a captured signal tracer, where SWV was used to measure the electrochemical response, achieving an LOD of 0.005 ng ml^−1^. Moazeni *et al*. [136] employed a stacked paper-based EI for AFP detection. The novel peptide-modified chip µPAD EI relies on impedimetric detection of AFP binding on the immunosensor surface. Diphenylalanine (FF) was used as a binding agent for the antibodies and for the sensing performance of the device. This peptide was used in the construction of the plastic-paper microfluidic chips, which consisted of a lower sheet of plastic and an upper sheet of chromatographic paper, modified with Ag-20 wt% graphene nanocomposite printed electrodes. The immunocomplex that resides on the Ag-graphene nanocomposite electrode is a simpler immunocomplex, as reviewed in a previous study. The immunocomplex is composed of FF nanorods with oxidized AFP-antibodies, where BSA was used to block binding sites so that the AFP biomarker was detected. This device only resulted in an LOD of 1 ng ml^−1^ and 10 ng ml^−1^ in PBS and plasma, respectively. These values were much higher than those reported in a previous study.

Cancer Antigen 125 (CA125) is a common tumour biomarker associated with ovarian cancer. Ovarian cancer is the leading cause of cancer-related deaths in women. Fan *et al*. [137] developed a µPAD EI for the detection of CA125 on Whatman No. 1 chromatographic paper using a screen-printing technique via DPV. A carbon counter and working electrode with an Ag/AgCl reference electrode were patterned on the paper substrate via screen printing using a simple template method. The working electrode was further modified with a reduced GO/thionine/AuNP nanocomposite (rGO/Thio/AuNP). This was performed to amplify the detection signal and immobilize the CA125 antibody. The rGO/Thio/AuNP nanocomposite was drop-coated onto the µPAD and annealed in an oven at 50°C for 15 min to increase the binding efficiency and remove the excess solvent. Following this, the CA125 antibody was immobilized on the nanocomposite to form an immunocomplex via strong non-covalent interactions between the amino groups of the antibody and AuNPs. As the concentration of CA125 increased, the current response of DPV decreased. This study produced a highly sensitive µPAD with an LOD of 0.012 ng ml^−1^. In a further study, Bahavarina *et al*. [138] developed a µPAD EI that detects CA125. In this study, a pen-on-paper technique was investigated for device fabrication. A platinum counter electrode with an Ag/AgCl reference electrode was used, unlike in the previously mentioned study. The working electrode had a similar modification as the previous study; however, it was modified to form a cysteamine-capped AuNP/Ag-rGO nanocomposite (CysA/AuNP/Ag-rGO) on the photographic paper. First, the Ag-rGO nano-ink electrode was drawn onto the surface of the paper, followed by RT drying for 5 min. Hydrazine hydrate was used for electrodeposition of CysA/AuNPs onto the surface of the Ag-rGO electrode, resulting in CysA/AuNP/Ag-rGO. To activate the CA125 antibody, EDC and NHS chemistry were used. Thus, immobilization of CA125 antibodies was achieved using positively charged amino group interactions. The LOD for this device was 0.94 ng ml^−1^, which is much higher than that reported by Fan *et al*. [137].

To date, lung cancer is one of the leading causes of malignant deaths worldwide. CEA is an excellent biomarker for this cancer and one of the most widely studied biomarkers. Wang *et al.* [139] produced a label-free µPAD EI that employed a screen-printed working electrode for the detection of CEA using DPV. Whatman No. 1 filter paper was used, where the working and counter electrodes were screen printed using Acheson carbon ink and a reference electrode with Ag/AgCl ink. The team prepared an amine-functionalized graphene–thionine–gold nanoparticle nanocomposite (NH_2_-GO/Thio/AuNP), which was then coated onto the working electrode of the SPCE. The CEA antibody was immobilized onto NH_2_-GO/Thio/AuNP to form an immunocomplex via the interaction of the amino group and AuNPs with the CEA antibody. The immunosensor was based on the decreasing current response of Thio being proportional to the CEA antigens present when forming an antibody–antigen immunocomplex. Overall, the team was able to produce a sensitive µPAD that achieved an LOD of 0.01 ng ml^−1^. Pavithra *et al*. [140] further developed a novel µPAD EI containing AuNP ink and derivate quinone for the detection of CEA using DPV. The working and counter electrodes were screen printed using the synthesized AuNP ink, whereas the reference electrode was printed using silver ink, after which they were cured in an oven. The amine and thiol groups were introduced onto the fabricated electrode by the addition of a mercapto-amine-functionalized receptor (R1). The purpose of this was to link the thiol and amino groups on R1 to the AuNP electrode surface and the CEA antigen. The µPAD used DPV for the detection of CEA and achieved an LOD of 0.33 ng ml^−1^. This is higher than that recorded in a previous study. Screen printing and drop-on-demand pen plotting are the most commonly employed paper-fabrication techniques. A range of cancer biomarkers were detected in the low ng ml^−1^ range. Graphene-gold nanocomposites are most commonly used for paper-based immunosensor fabrication. The bulk of the work suggested for cancer detection has been conducted in label-free configurations relying on voltammetric detection in the presence of redox probes.

### 6.3. µPED immunosensors for bacteria

Bacteria are single-celled organisms known for their resilience, aided by unique enzymes (extremozymes) that allow them to survive in extreme conditions of high pressures and temperatures [179]. In the human body, various benign bacteria play vital roles in bodily functions [180]. However, specific bacterial species can lead to illnesses and fatalities, such as *Campylobacter, Clostridium perfringens, Escherichia coli, Listeria monocytogenes, Pseudomonas aeruginosa, Salmonella typhimurium* and *Staphylococcus aureus* to name just a few. Detecting pathogens that cause microbial infection outbreak is pivotal [181]. Research in the field of bacteria sensing has far-reaching benefits in green energy development [182], pharmaceutical manufacturing [183], research into health products and environmental control/monitoring [184]. While traditional techniques for the identification of bacteria including Gram staining [185], and culture and biochemical methodologies [186] have been invaluable, they do have limitations.

Research has been conducted on bacterial detection for food and environmental control using various methods for different applications [187–189]. *S. aureus* [143,190,191], *S. typhimurium* [144,192,193], *Microcystin-LR* [145,194], *E. coli* [195–197], *L. monocytogenes* [198,199] and *P. aeruginosa* [200–202] are popular pathogens that can be fatal to humans and animals. Bhardwaj *et al*. [143] developed a label-free µPAD EI for the detection of *S. aureus* using an antibody- (Ab-) single-walled carbon nanotube (SWCNT) bio-conjugate with DPV. To reduce the number of immunoassay processing steps, anti-*S. aureus* antibodies (Abs) were covalently bonded to SWCNT using EDC/NHS as a coupling reagent. The bio-conjugate, Ab-SWCNT, was then immobilized onto a Whatman No. 3 chromatography paper stencil-printed electrode, where DPV was used to detect *S. aureus* via the change in peak current after the formation of the antigen–antibody complex. The biosensor was tested against *E. coli, B. subtilis* and *S. epidermidis*; however, it was observed that the sensor was specific only to *S. aureus*. The µPAD EI developed by Bhardwaj *et al*. was sensitive and specific, achieving an LOD of 13 CFU/ml. Silva *et al*. [144] proposed a µPAD potentiometric immunosensor for the detection of *S. typhimurium* that relies on blocking surface principles. Two different immunosensing methods were applied to the filter paper-strip electrodes. The first method used direct conjugation of the antibody to the polymer membrane, and the second method used conjugation of antigen–dendrimer coupling. As before, this study used EDC/NHS chemistry to activate the electrode surface for successful immunocomplex building. To assess the modification and analytical performance of the immunosensor, EIS was conducted at an LOD of 5 cell/ml. Yao *et al*. [145] developed a conductive nanobiochar µPAD EI for the detection of *microcystin-LR* toxins in water. The paper-based immunosensor was constructed by coating conductive nanobiochar particles and an anti-microcystin-LR antibody on filter paper via a dip-and-dry method. This study used amperometry for the detection of *microcystin-LR* where the biosensor had a response within 5 min with an LOD of 0.017 µg l^−1^. Additionally, this device is highly selective and reproducible, with a great storage stability.

### 6.4. µPED EIs for viruses

Viruses are tiny infectious agents consisting of genetic material enclosed in a protein coat [203]. They need a host to multiply, making them inherently pathogenic. HIV alone has claimed 36 million lives [204], highlighting the urgency of virus detection and monitoring. Deadly viruses include Marburg, Ebola, rabies, HIV, influenza, hantavirus, smallpox, dengue, rotavirus and coronaviruses.

Research on µPAD EIs for accurate, inexpensive and sensitive detection using small sample sizes is being conducted to reduce the risk of accidental transmission and establish sampling requirements. In recent years, paper-based EIs have become popular choices for POC analytical devices. Currently, there are devices for the detection of influenza [146,147], hepatitis [148,205,206] and HIV [205]; however, there is little to no work in this field. Devarakonda *et al*. [146] developed a label-free µPAD EI to detect influenza virus H1N1 using DPV to assess the performance of the biosensor. Whatman chromatography No. 4 paper was modified with silica nanoparticles to introduce hydrophobicity and stencil-printed electrodes. To increase the sensitivity of the sensor, the stencil-printed electrodes were modified with SWCNT and chitosan, and the antibodies were immobilized using glutaraldehyde cross-linking. The device produced was sensitive and selective, as it was tested against MS2 bacteriophages and influenza B viruses, obtaining an LOD of 113 PFU/ml for onsite analysis of influenza H1N1 viruses. Multiplexed detection devices have become increasingly popular in recent years. Another study on the influenza virus was conducted by Lee *et al*. [147], who developed a label-free µPAD EI to detect multiple avian influenza virus antigens, namely H5N1, H7N9 and H9N2, via the DPV electrochemical technique. This work is unique in that it addresses the physical weakness of µPADs when wet by introducing flexible screen-printed electrodes modified by carbon nanotube-polydimethylsiloxane. To activate the –COOH groups that functionalize the CNTs, EDC/NHS chemistry was used to immobilize the respective haemagglutinin antibodies onto the electrodes. As stated previously, DPV was used to detect changes in the electrochemical response upon the formation of antigen–antibody complexes. The LODs obtained for H5N1, H7N9 and H9N2 haemagglutinin antigens were 55.7, 99.6 and 54 pg ml^−1^, respectively. A more advanced and modern approach for virus detection was demonstrated by Boonkaew *et al*. [148], who developed a stacked orientated sequential µPAD EI for the simultaneous detection of the hepatitis B surface and hepatitis C core antigens. The delivery of the immunosensing fluid was controlled by the integration of fast-flowing and delaying flow behaviours via the stacking orientation. This is unique because the fast-flowing channel was used as an automated system that washes the unbound antigens, whereas the delayed channels were used to store redox reagents for electrochemical analysis using chronoamperometry. This µPAD electrochemical immuno-biosensor was able to achieve LODs of 18.2 and 1.19 pg ml^−1^ for hepatitis B surface antigen and hepatitis C core antigens, respectively.

### 6.5. µPED immunosensors for SARS-CoV-2

SARS-CoV-2 is a respiratory disease that forms part of the coronavirus family [207]. It is spread through aerosol droplets, resulting in over 6 million deaths [208]. Molecular, antibody and antigen testing in paper-based substrates are common.

Over the past 4 years, research has been conducted on various SARS-CoV-2 electrochemical biosensors. These include immunosensors [209–212] and nucleic acid sensors [213–215]. Although they are accurate, sensitive and rapid, they are still inexpensive. Research into µPAD EIs for SARS-CoV-2 is rare. Advances in this field are new, and only a limited amount of research has been conducted on it. This indicates a gap in the field of knowledge.

Ehsan *et al*. [149] produced a label-free µPAD EI for the detection of the SARS-CoV-2 RBD spike protein (S-protein) using electrochemical impedance spectroscopy. The team used a screen-printed technique to fabricate cellulose-fibre-based pads, which were eventually cut into strips. To increase the conductivity of the immunosensor, a graphene/carbon hybrid ink was used as the electrodes. This was because the team aimed for high conductivity, and impedance-based sensors need to have an extended detection range by providing sharper and low baseline impedance and increasing sensitivity. The biosensor uses the IgG anti-SARS-CoV-2 spike antibody, which is immobilized onto a graphene/carbon electrode. Owing to the high sensitivity of the impedance electrochemical technique, this µPAD could achieve an LLOQ of 0.25 fg ml^−1^. Another SARS-CoV-2-specific RBD S-protein was developed, in which a paper-based immunosensor using a plant-based anti-SARS-CoV-2 monoclonal antibody CR3022 was immobilized onto the cellulose nanocrystal-modified electrode surface. The purpose of the cellulose nanocrystal modification of the electrode was to provide abundant –COOH functional groups to encourage higher antibody immobilization. Using DPV, the 3D folding paper-based immunosensor designed by Jaewjaroenwattana *et al.* [150] achieved an LOD of 2.0 fg ml^−1^. Yakoh *et al*. [151] developed a label-free µPAD EI for the detection of SARS-CoV-2-specific IgG and IgM antibodies against SAR-CoV-2 using SWV. Screen-printed electrodes were printed on Whatman No. 4 chromatography paper using a GO-modified electrode for an origami-orientated device. GO was strategically used as it contains –COOH groups that could be activated by EDC and NHS chemistry, resulting in excellent immobilization of the S-protein. What makes this research unique is that the S-protein was immobilized onto the GO-modified electrode and detected for SARS-CoV-2-specific IgG and IgM antibodies. In the presence of immunoglobulins, the redox reaction is disrupted, resulting in a decreased current response in the SWV analysis. The approach to this µPAD produced a specific and sensitive EI, which resulted in LODs of 0.96 and 0.14 ng ml^−1^ for IgG and IgM, respectively. A trend can be seen in these studies, as both use graphene-based electrodes.

In the light of the many advantages of electrochemical paper-based biosensors, Ali Farzin *et al.* [152] developed a voltammetric genosensor for the detection of the SARS-CoV-2 RdRP gene via a cDNA probe/Au@CD core–shell NP/graphite nanocrystal/paper electrode. This is the first time that graphite nanocrystals have been employed in electrochemical biosensors. Using DPV to measure the change in the reduction peak current, the nanomaterial-modified paper genosensor was exposed to different concentrations of SARS-CoV-2 genes, and the hybridization between cDNA and the RdRP gene was monitored using the toluidine blue redox probe. The response of the genosensor exhibited great stability because of the strong adhesion of the graphite nanocrystal layer on the paper substrate, as well as the strong bonding affinity of the cDNA-Au@CD bio-conjugates, achieving an LOD of 0.15 pM. With the increasing development of sensing technology, smartphones can now be incorporated to assist with diagnostic tools. As discussed previously, Lomae *et al.* [153] developed a smartphone-assisted paper-based electrochemical genosensor for the detection of SARS-CoV-2. The premise of this genosensor is to modify the working electrode with acpcPNA, which is a biorecognition element that binds via hydrogen bonding to the cDNA. The hybridization of cDNA and acpcPNA results in a blockage of the redox reaction, in which the electrochemical response decreases in relation to the SARS-CoV-2 concentration. Using amperometric electrochemistry, the fabricated paper-based genosensor achieved an LOD of 1 pM.

### 6.6. Other µPED immunosensors

Many other µPAD EIs are useful in modern society. Advances in this field have led to the development of µPADs for infection/inflammation monitoring. C-reactive proteins were detected at graphene/AuNP functionalized origami paper-based electrochemical immunoassays and disposable thiol-terminated polymers, respectively [154,155]. For its role in the diagnosis of tuberculosis, human interferon-gamma was studied on folded impedimetric paper-based devices functionalized with polyaniline-graphene films [156]. In a separate study, multi-walled carbon nanotube nanocomposites were employed to monitor the oestradiol hormone on paper-based devices [157]. Multi-walled carbon nanotubes were further applied to detect aflatoxin B1 (AFB1), a fungal detection in food, using an impedimetric paper-based device [158]. Along with these, many other protein biomarkers for disease detection are being proposed.

**Figure 7.**
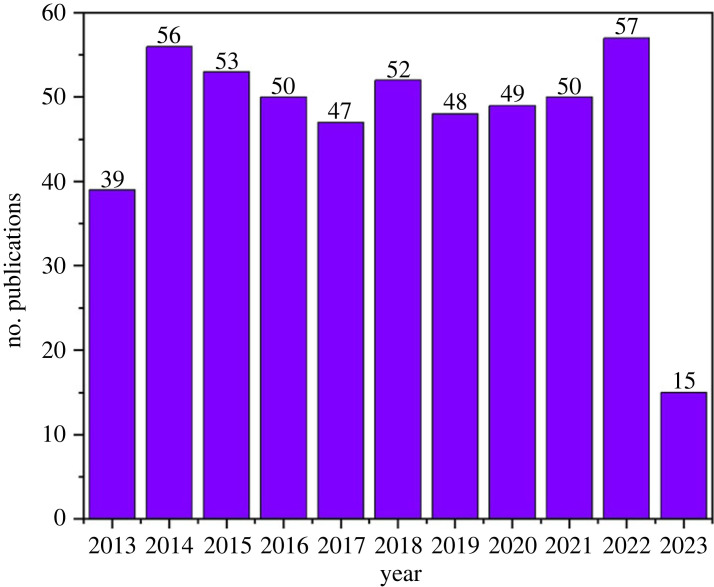
Bar graph showing the number of publications in the research field of EIs extracted from Scopus.

In table 2, several consistent trends emerge across the various types of µPEDs for the detection of analytes. Whatman filter paper, particularly Whatman No. 1, is the favoured substrate for sensor fabrication due to its balanced retention and flow rate, which are advantageous for sensing. Screen printing is the preferred method for electrode fabrication, complemented by wax printing to establish hydrophobic barriers and hydrophilic channels. This combined approach significantly enhances fluid control, which in turn improves the sensitivity of the sensor. DPV emerges as the popular choice for the electrochemical detection of analytes due to its intrinsic lower capacitive current with sensitivity surpassing the capabilities of CV. Detection was achieved in the femtogram to nanogram per millilitre range. Notably, graphene and other carbon nanomaterial-based electrodes, often paired with metallic nanomaterials, are extensively employed to heighten sensitivity of the paper electrode substrates. Consequently, µPEDs using graphene-based electrodes have the lowest limits of detection. Figure 7 illustrates the yearly publication count over the last decade in the field of EIs, based on Scopus data. On average, around 50 research papers dedicated to EIs are published annually, underscoring the ongoing development in this field of research.

### 6.7. Future prospects in µPAD EIs for SARS-CoV-2

Tremendous strides in fabrication techniques have led to a rapid increase in the use of paper-based microfluidics in energy and diagnostic tools. The rapidly expanding field has seen improvement in paper patterning with excellent resolution creating features in the low µm range in some cases. Moreover, innovative fluid control has expanded its use to previously inaccessible ranges. This growth has seen its use skyrocket for colorimetric, electrochemical and electronic sensing of biomedical and environmental fields. However, limitations in sensitivity and reliability of electrochemical paper-based devices are crucial for their widespread commercialization. These devices have generally shown improved detection capabilities over optical paper-based systems where observable changes for low concentrations often go unnoticed. Moreover, issues related to power supply further limit the use of electrochemical diagnostics. Although advances have been made in the field of µPADs for biomedical applications, the detection of new diseases such as SARS-CoV-2 creates new gaps in research. According to our data, approximately 57% of the Earth's population has been fully vaccinated [216]. That said, it leaves opportunities and a need for research to be directed toward the efficacy of the SARS-CoV-2 coronavirus vaccine. There has been no work done on paper-based SARS-CoV-2 EIs using graphene quantum dots (GQD), which are advantageous for EIs. GQD are excellent electron transporters, as they can expand their surface area when in contact with an analyte, making them more electrochemically active [217]. Additionally, they contain carboxyl, hydroxyl and carbonyl functional groups owing to their biocompatibility, which allows for the immobilization of biological analytes [218,219]. This provides an excellent candidate for use of GQD in a µPAD SARS-CoV-2 EI. GQD and also nanomaterials as a whole bring many benefits to the biosensing world in the fabrication of immunosensors. Future µPADs will continue to make use of nanomaterials to enhance the sensitivity and selectivity of devices. They do so either by modifying the solid support in heterogeneous sensing devices, or as labels on antibodies and antigens. The incorporation of smartphones into signal measurement will become more common in this research area as they are widely available and have the computing capacity to analyse signals. An example is a programme/application on a smartphone that can be developed to analyse all types of electrochemical plots, such as voltammograms or DPV graphs. Overall, the development of µPADs for biosensing purposes has grown significantly in recent years and will be a staple in creating cheaper alternative diagnostic tools as new diseases emerge over time. Particularly useful are the advancements made in fluid separation and handling. The field offers great prospects for commercialization as a rapid technique for diagnosis, but significant improvements are required for widespread use, particularly in the medical community.

## Data Availability

All research data can be found within this publication.
